# Thoracic perfusion of recombinant human endostatin (Endostar) combined with chemotherapeutic agents versus chemotherapeutic agents alone for treating malignant pleural effusions: a systematic evaluation and meta-analysis

**DOI:** 10.1186/s12885-016-2935-4

**Published:** 2016-11-14

**Authors:** Rong Biaoxue, Cai Xiguang, Liu Hua, Gao Wenlong, Yang Shuanying

**Affiliations:** 1 Department of Respiratory Medicine, First Affiliated Hospital, Xi’an Medical University, 48 Fenghao West Road, Xi’an, 710077 China; 2 Department of Respiratory Medicine, Gansu Provincial Hospital, Lanzhou, China; 3 Department of Statistics and Epidemiology, Medical College, Lanzhou University, Lanzhou, China; 4 Department of Respiratory Medicine, Second Affiliated Hospital, Xi’an Jiaotong University, Xi’an, China

**Keywords:** Endostar, Malignant pleural effusions, MPE, Meta-analysis, Efficacy, Safety

## Abstract

**Background:**

Endostar is a new endogenous angiogenic inhibitor with implicated anti-tumor activity. This study was to investigate whether thoracic perfusion of Endostar could be used to control malignant pleural effusions (MPE).

**Methods:**

We searched the databases of MEDLINE, Web of Science, EMBASE, Goggle, Cochrance Library and CNKI to select the studies regarding the efficacy of Endostar to treat MPE. A total of 13 randomised controlled trials (RCTs) with 1066 patients were included.

**Results:**

The overall response rate (ORR) (*P* < 0.001; odds ratio = 3.58) and disease control rate (DCR) (*P* < 0.001; odds ratio = 2.97) of Endostar combined with chemotherapeutic agents were significantly higher than those of chemotherapeutic agents alone. In addition, Endostar combined treatment remarkably promoted quality of life (QOL) of patients (*P* < 0.001; odds ratio = 3.04) compared with that of chemotherapeutic agents alone. Moreover, Endostar combined treatment did not have an impact on the incidence of adverse reactions (AEs) (*P* < 0.05).

**Conclusions:**

The efficacy of Endostar combined chemotherapeutic agents was superior to chemotherapeutic agents alone through thoracic perfusion in treating MPE, which indicated that Endostar could be an effective agent for controlling MPE.

## Background

In China, lung cancer has been becoming a major cause of death in malignant tumors due to increasingly air pollution and deterioration of the natural environment. In 2015 in China, There is dreadful fact that 733,000 lung cancer cases were diagnosed and 610,000 patients will die from this disease [1]. In clinic, most of lung cancer patients always are accompanied with the event of malignant pleural effusions (MPE), which leads to a lower quality of life and even reduced the life expectancy. Thus, doctors often pay more attention to the treating of MPE and the prolongation of survival [2]. Traditional treatments of MPE include drainage of pleural effusion, pleural adhesions and pharmacotherapy and so on. In addition, thoracic perfusion of chemotherapeutic agents has been suggested to be used in controlling of the effusion. The main opinion is that the intrapleural levels of a chemotherapy agent administered into the pleural space can be significantly higher than the systemic levels [3]. However, most of lung cancer cases are often resistant to standard chemotherapy agent, or eventually become chemoresistant. Therefore, the fact is many chemotherapeutic agents are not as effective as we expected in treating MPE via thoracic perfusion [4]. Today, novel molecular targeted drugs that have been studied to improve the cure and control rate of the disease. Because of strong antineoplastic activity and low toxicity, these products have been used as alternative treatments for the control of MPE [5].

Endostatin is a naturally-occurring, 20-kDa C-terminal fragment derived from type XVIII collagen, which was first reported by Folkman. Endostatin has been reported to inhibit angiogenesis in a wide range of tumors, and may interfere with the pro-angiogenic effects of growth factors. Capillary endothelial cells are the targets of endostatin, endostatin blocks endothelial cell proliferation and formation of new blood vessels, and affects the progress and metastasis of malignant tumors [6]. A new recombinant human endostatin (code number: YH-16), Endostar, is developed by Simcere-Medgenn Bioengineering Co. Ltd, Nanjing and Yantai, China, and is different from the original endostatin studied by O’Reilly [6, 7], which was approved by the China State Food and Drug Administration (SFDA) for the treatment of non- small cell lung cancer as the first therapy in 2005 [8]. Endostar has a structural difference compared with endostatin reported in previous literature, which purified in Escherichia coli with an additional nine-amino acid sequence (MGGSHHHHH) [9, 10]. Some studies suggested that the antiangiogenic biological function has been promoted because of such a structural changes on this drug in treating lung cancer [8, 11, 12].

Recent years, some studies have specially investigated the clinical effect and safety of Endostar combined with chemotherapeutic agents versus chemotherapeutic agents alone in treating MPE via thoracic perfusion. Here, we performed a systematic literature review to assess the clinical benefit of Endostar combined therapy in treating MPE.

## Methods

### Identification of literature

We searched and identified relevant randomized controlled trials (RCTs) from the databases of MEDLINE/PubMed, EMBASE, Cochrance Library, SCI, and CNKI database (from January 2005 to April 2016). We adopted various MeSH terms and key words that related to MPE and Endostar as follows: “malignant pleural effusion,” “MPE,” “rh-endostatin,” “endostatin,” “chemotherapy,” “Endostar,” and “recombinant human endostatin injection.” In addition, if we found useful information that was intimately associated with Endostar in the reference lists of those studies, we should further look for additional studies and identified them.

### Collection of study variables

The data that we extracted included: (1) the number of patients of each RCT, (2) publication date of literature, (3) the clinical characteristics of data, (4) the ways of clinical intervention, (5) overall response (ORR) and disease control rate (DCR) and (6) quality of life (QOL) and adverse effects (AEs).

### Criteria that studies were included and excluded

Inclusion criteria: (1) studies must be designed to compare Endostar plus chemotherapeutic agents to chemotherapeutic agents alone; (2) patients must be given drugs through thoracic perfusion; (3) patients must be diagnosed with MPE; (4) outcome measures must be reported; and (6) the total cases of patients must be greater than or equal to (but not less than) 50. Exclusion criteria: (1) studying on animals not human; (2) patients were given excessive other adjutant drugs; (3) studies were sponsored by pharmaceutical manufacturers; and (4) study was short of efficient control group.

### Supervision of the implementation process

Trial design: RCTs of Endostar combined with chemotherapeutic agents versus chemotherapeutic agents alone through thoracic perfusion for treating MPE. The ways of interventions: the dosage was defined according to the statement of manufacturers and frequency of administration at least 2 times; Evaluation indicators of therapeutic efficacy: ORR, DCR, QOL, and AEs.

### Quality assessment of included RCTs

We utilized the evaluation criteria shaped by Cochrane Handbook (Version 5.0.1) to assess the included trials, which included: (1) methods to random group of patients; (2) how to perform an adequate setting blinding; (3) how to perform an adequate allocation and conceal the sequence; and (4) a description of intention to treat. Eventually, the quality of trials was divided into three levels: low risk of bias, unclear risk of bias, and high risk of bias [13, 14].

### Statistical methods and analysis

After sufficient data were collected and identified, the process of meta-analysis was implemented. The odds ratio (OR) with 95 % confidence intervals (CI) were major statistical data that were applied to explore the difference of efficacy. The overall effect was calculated by Z-scores and *P*-values <0.05 were considered to be statistically significant. The identification of homogeneity was studies was calculated by the *χ* ^2^ statistic and was quantified with the I^2^ statistic. In our study, we adopted fixed effects model preferential to perform meta-analysis. We also used meta-regression to evaluate whether the results were different between two groups. In order to assess the bias of literature, we omitted each study from the estimated pool to analyze the influence of each study to overall effect. In addition, funnel plots, Egger’s test, and Begg’s test were performed to assess publication bias. We used SPSS (SPSS Institute, version 19.0, Chicago, USA) and Stata version 15.0 (Stata Corporation, TX, USA) to implement the statistical analysis and used a significance level of *P* <0.05.

## Results

### Study selection process

**Fig. 1:**
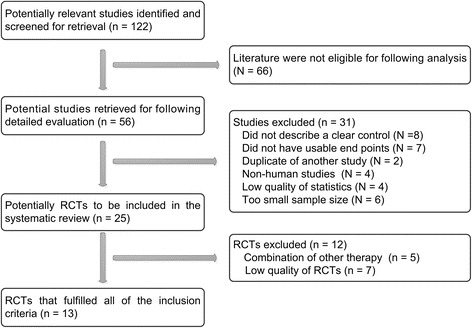
Flow chart of literature search. RCTs, randomized controlled trials

Originally, 122 potentially relevant studies were identified. Of them, 66 studies were removed because they were not original literature such as review, abstract and meeting records. Remaining 56 studies were identified as requiring RCTs, but 31 studies were excluded subsequently because of the following reasons: did not describe a clear control; did not have usable end points; duplicate of another study; non-human studies; low quality of statistics; and too small sample size. Of the remaining 25 trials, 12 were excluded further because complicated combination therapy and low design quality. Finally, 13 [15–27] studies published between 2010 and 2015 were included (Fig. 1). The eligible studies contained a total of 1066 patients with the sizes of distributing from 56 [18] to 120 [20] patients. The cause of MPE mainly included lung cancer (918 cases), breast cancer (78 cases) and digestive tract tumors (56 cases). A meta-analysis database was established according to the extracted data, which was listed in Tables 1 and 2.

**Table 1:** Data analysis of included studies

Study	*N*	Male	Female	Age (average)	Sources of tumor (N)				Volume of MPE (*N*)	Quality of Life	End point
					Lung/pleura	Breast	Digestive tract	Others			
Liu W 2010 [15]	96	51	45	40–70	96	–	–	–	Unavailable	KPS	RR, DCR, SI, AEs
Mao L 2011 [16]	90	45	45	27–70	81	5	2	2	>1000 ml	KPS	RR, DCR, SI, AEs
Li G 2011 [17]	60	30	30	41–76	25	20	15	–	Large (37) Moderate (23)	KPS	RR, DCR, SI, AEs
Ma E 2012 [18]	56	32	24	35–67	56	–	–	–	>1000 ml	Unavailable	RR, DCR, SI, AEs
Yao Q 2012 [19]	60	42	18	35–78	28	16	4	12	>1000 ml	KPS	RR, DCR, SI, AEs
Zheng Q 2013 [20]	120	73	47	32–75	78	25	17	–	>1000 ml	KPS	RR, DCR, SI, AEs
Kang Y 2013 [21]	90	53	37	18–72	90	–	–	–	>1000 ml	KPS	RR, DCR, SI, AEs
Wen J 2014 [22]	60	34	26	35–65 (50.5)	45	9	6	–	Large (13) Moderate (29) Small (18)	KPS	RR, DCR, SI, AEs
Yue G 2014 [23]	86	48	42	38–69	86	–	–	–	Unavailable	KPS	RR, DCR, SI, AEs
Tu J 2014 [24]	90	41	49	45–70	90	–	–	–	Unavailable	KPS	RR, DCR, SI, AEs
Xu J 2014 [25]	70	43	27	44–70	70	–	–	–	>1000 ml	KPS	RR, DCR, SI, AEs
Wen X 2015 [26]	104	69	35	39–76	104	–	–	–	Unavailable	KPS	RR, DCR, SI, AEs
Hu X 2015 [27]	84	62	22	18–70	69	3	12	–	>1000 ml	ECOG	RR, DCR, SI, AEs

*N* number of patients, *MPE* malignant pleural effusion, *KPS* karnofsky physical status score, *RR* response rate, *DCR* disease control rate, *SI* symptom improvement, *AEs* adverse effects, *ECOG* Eastern Cooperative Oncology Group (performance status)

**Table 2:** Assessment of administration of included studies

Study	Trial group (*N*)	Control group (*N*)	Interventions		Treatment cycle	Termination of treatment
			Endostar combined with chemotherapeutic agents	Chemotherapeutic agents alone		
Liu W 2010 [15]	32	32	Cisplatin 40 mg/m^2^, 1/week Endostar 30 mg, 1/week	Cisplatin 40 mg/m^2^, 1/w	3 weeks	>3 weeks, or pleural effusion disappeared
Mao L 2011 [16]	45	45	Cisplatin 40 mg/m^2^, 1/week Endostar 30 mg, 2/week	Cisplatin 40 mg/m^2^, 1/7d	7d/cycle, 2 cycles	>4 cycles, or pleural effusion disappeared
Li G 2011 [17]	30	30	Bleomycin 60 mg, 1/week Endostar 30 mg, 1/week	Bleomycin 60 mg, 1/w	3 weeks	>3 weeks, or pleural effusion disappeared
Ma E 2012 [18]	28	28	Cisplatin 40 mg/m^2^, 1/week Endostar 30 mg, 1/week	Cisplatin 40 mg/m^2^, 1/w	4 weeks	>4 weeks, or pleural effusion disappeared
Yao Q 2012 [19]	30	30	Nedaplatin 40 mg, 1/week Endostar 45 mg, 1/week	Nedaplatin 40 mg, 1/w	4 weeks	>4 weeks, or pleural effusion disappeared
Zheng Q 2013 [20]	60	60	Cisplatin 30–40 mg, d1–3 Endostar 90 mg, d4	Cisplatin 30–40 mg d1–3	21d/cycle, 1–4 cycles	>4 cycles, or pleural effusion disappeared
Kang Y 2013 [21]	45	45	Cisplatin 40 mg, 2/week Endostar 30 mg, 2/week	Cisplatin 40 mg, 2/w	3 weeks	>3 weeks, or pleural effusion disappeared
Wen J 2014 [22]	25	29	Lobaplatin 30 mg/m^2^, d1 Endostar 30 mg, d1	Lobaplatin 30 mg/m^2^, d1	4 cycles	>4 cycles, or pleural effusion disappeared
Yue G 2014 [23]	43	43	Cisplatin 60 mg, 1/week Endostar 30 mg, 2/week	Cisplatin 60 mg, 1/w	2–3 weeks	>4 weeks, or pleural effusion disappeared
Tu J [24]	45	45	Cisplatin 40 mg/m^2^, 2/week Endostar 45 mg, 2/week	Cisplatin 40 mg/m^2^, 2/w	3 weeks	>3 weeks, or pleural effusion disappeared
Xu J 2014 [25]	35	35	Nedaplatin 60 mg, 1/week Endostar 60 mg, 1/week	Nedaplatin 60 mg, 1/w	4 weeks	>4 weeks, or pleural effusion disappeared
Wen X 2015 [26]	68	36	Bleomycin 45 mg, w1 Endostar 45 mg, w2	Bleomycin 45 mg, 1/7d	7d/cycle, 2–4 cycles	>2 weeks, or pleural effusion disappeared
Hu X 2015 [27]	43	41	Cisplatin 40 mg, 2/week Endostar 60 mg, 2/week	Cisplatin 40 mg, 2/week	2 weeks	>2 weeks, or pleural effusion disappeared

*N* numbers of patients, *d* day, *w* week

### Quality of study design

The design of 8 studies were that Endostar combined with cisplatin versus cisplatin alone through thoracic perfusion for treating MPE [15, 16, 18, 20, 21, 23, 24, 27], 2 studies were Endostar combined with bleomycin versus bleomycin alone [17, 26], 2 studies Endostar combined with nedaplatin versus nedaplatin alone [19, 25] and one Endostar combined with lobaplatin versus lobaplatin alone [22]. The dosage of Endostar via thoracic perfusion and follow-up times for efficacy evaluation had a good consistency, which was shown in Table 2. Generally, the dosage of Endostar was administered at the range of 30–90 mg per one time and frequency of administration was two times at least, which were dissolved in physiological saline, and given by thoracic perfusion after drainage of pleural effusions.

### Quality of study characteristics

Two investigators independently reviewed and determined the quality of each study. The discrepancies were resolved by consensus with the third expert. The evaluation was performed according to the evaluation criteria established by Cochrane Handbook (Version 5.0.1) [28]. As shown in Table 3, the results showed that 4 of the 13 studies (30.7 %) belonged to the low risk of bias [17, 20, 24, 27], and the rest [1, 4, 5, 8–10, 15, 16, 18, 19, 21–23, 25, 26, 29] were inclined to indicate an unclear risk of bias (69 %) (Table 3).

**Table 3:** Design quality of included trials

Studies	Region	Sequence generation	Allocation concealment	Blind	Outcome data	Selective outcome reporting	Other sources of bias	ITT	Risk of bias
Liu W 2010 [15]	Single center	Random number table (SPSS)	Clear	Clear	Yes	No	Clear	No	Unclear risk of bias
Mao L 2011 [16]	Single center	Random number table (SAS)	Unclear	Unclear	Yes	No	Clear	Yes	Unclear risk of bias
Li G 2011 [17]	Single center	Random number table (SAS)	sufficient	Unclear	Yes	No	Clear	Yes	Low risk of bias
Ma E 2012 [18]	Single center	Random number table (SPSS)	Unclear	Unclear	Yes	No	Clear	No	Unclear risk of bias
Yao Q 2012 [19]	Single center	Random number table (SPSS)	Unclear	Unclear	Yes	No	Clear	No	Unclear risk of bias
Zheng Q 2013 [20]	Single center	Random number table (SAS)	sufficient	Unclear	Yes	No	Clear	Yes	Low risk of bias
Kang Y 2013 [21]	Single center	Random number table (SAS)	Unclear	Unclear	Yes	N0	Clear	Yes	Unclear risk of bias
Wen J 2014 [22]	Single center	unclear	Unclear	Unclear	Yes	N0	Clear	Yes	Unclear risk of bias
Yue G 2014 [23]	Single center	Random number table (SAS)	Unclear	Unclear	Yes	No	Clear	Yes	Unclear risk of bias
Tu J 2014 [24]	Single center	Random number table (SPSS)	Insufficient	Unclear	Yes	No	Unclear	Yes	Low risk of bias
Xu J 2014 [25]	Single center	Random number table (SPSS)	Unclear	Clear	Yes	No	Unclear	No	Unclear risk of bias
Wen X 2015 [26]	Single center	Random number table (SPSS)	Unclear	Unclear	Yes	No	Clear	No	Unclear risk of bias
Hu X 2015 [27]	Single center	unclear	Insufficient	Unclear	Yes	No	Clear	No	Low risk of bias

*SAS* SAS software, *SPSS* SPSS software, *ITT* intention-to-treat

### Comparison of ORR

**Fig. 2:**
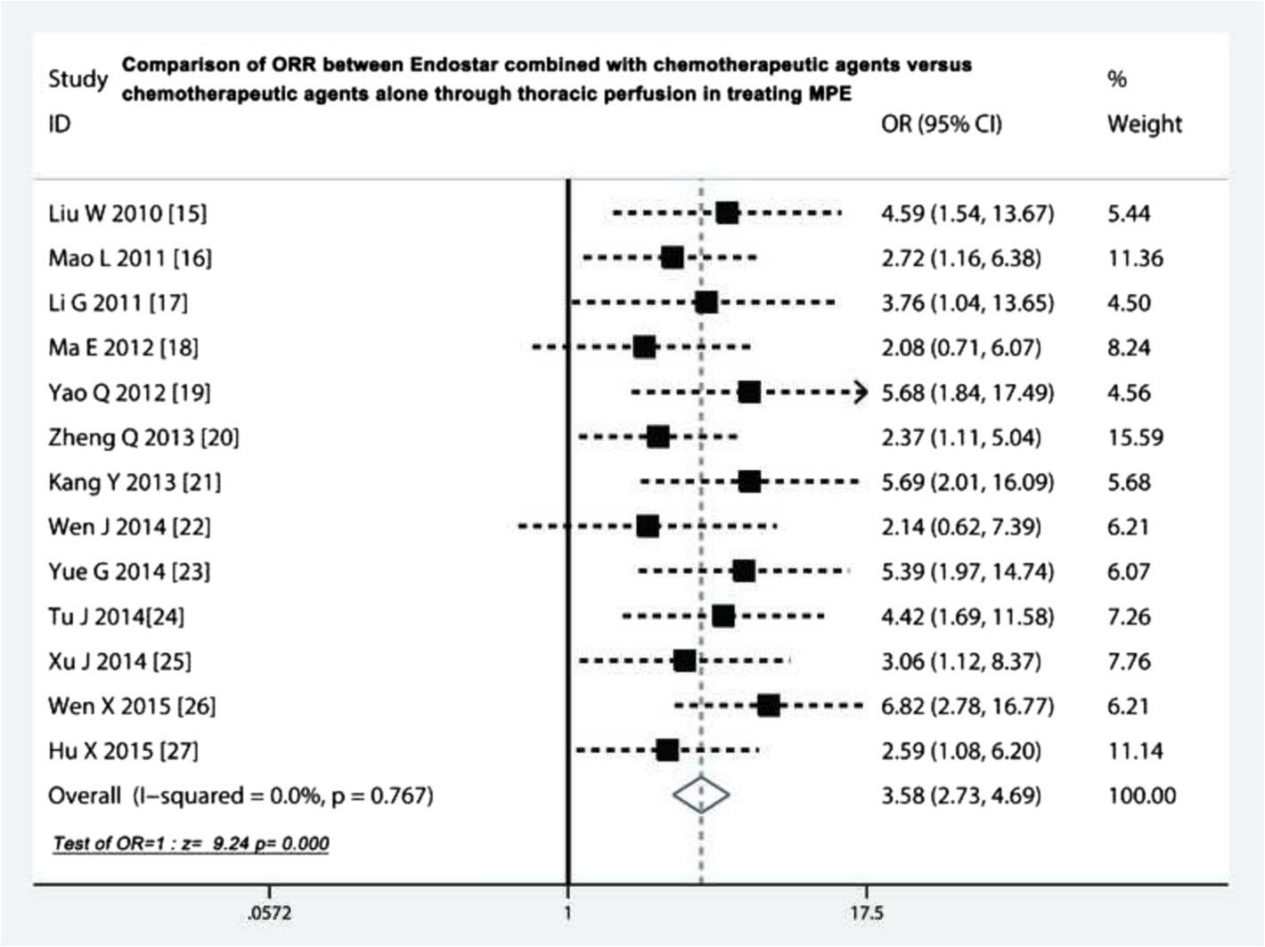
Comparison of ORR between Endostar combined with chemotherapeutic agents versus chemotherapeutic agents alone through thoracic perfusion for treating MPE. ORR, overall response rate; OR, odds ratio; MPE, malignant pleural effusions

We identified 13 RCTS [15–27] pertaining to ORR comparison. The odds ratio of fixed-effects was 3.58 (95 % CI 2.73 to 4.69; Z = 9.24, *p* < 0.001; Fig. 2), which indicated that the ORR of Endostar combined treatment was significantly higher than that of chemotherapeutic agents alone. Among these 13 studies, we did not observe the evidence of heterogeneity (heterogeneity chi-squared = 8.23; *p* = 0.767). Moreover, sensitivity analysis revealed the odds ratio and 95 % CI did not change when we omitted anyone study, with an odds ratio pool changing between 2.08 to 6.82.

### Comparison of DCR

**Fig. 3:**
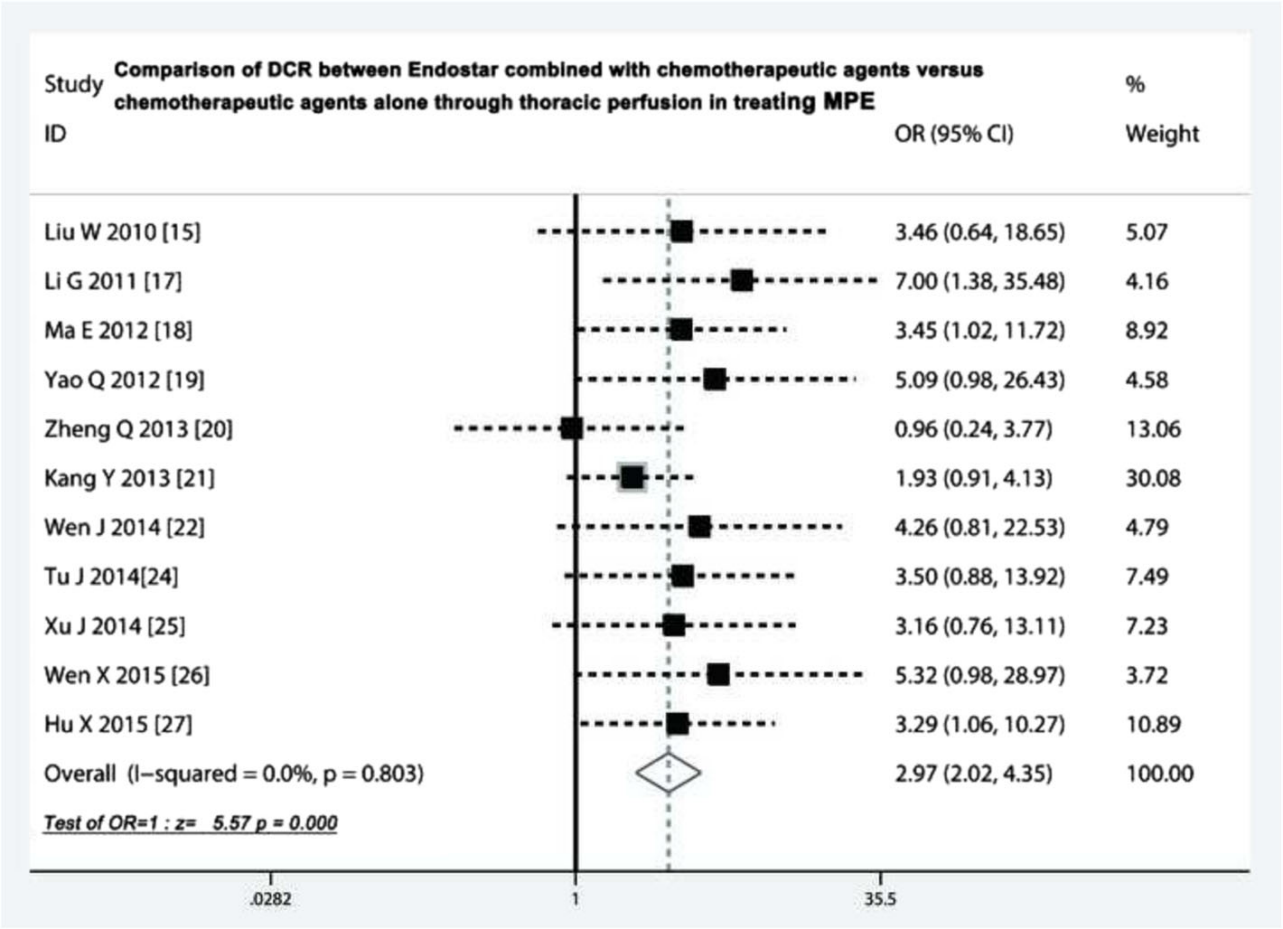
Comparison of DCR between Endostar combined with chemotherapeutic agents versus chemotherapeutic agents alone through thoracic perfusion for treating MPE. DCR, disease control rate; OR, odds ratio; MPE, malignant pleural effusions

Eleven trials [15, 17–22, 24–27] compared the DCR. The odds ratio of the fixed effects model ranged from 0.96 to seven and did not imply the existence of heterogeneity (heterogeneity chi-squared = 6.15; *p* = 0.803). The pooled odds ratio was 2.97 (95 % CI 2.02 to 4.35; Z = 5.57, *p* < 0.001), which indicated that Endostar combined with chemotherapeutic agents promoted the DCR, compared with chemotherapeutic agents alone (Fig. 3).

### Comparison of QOL after treatment

**Fig. 4:**
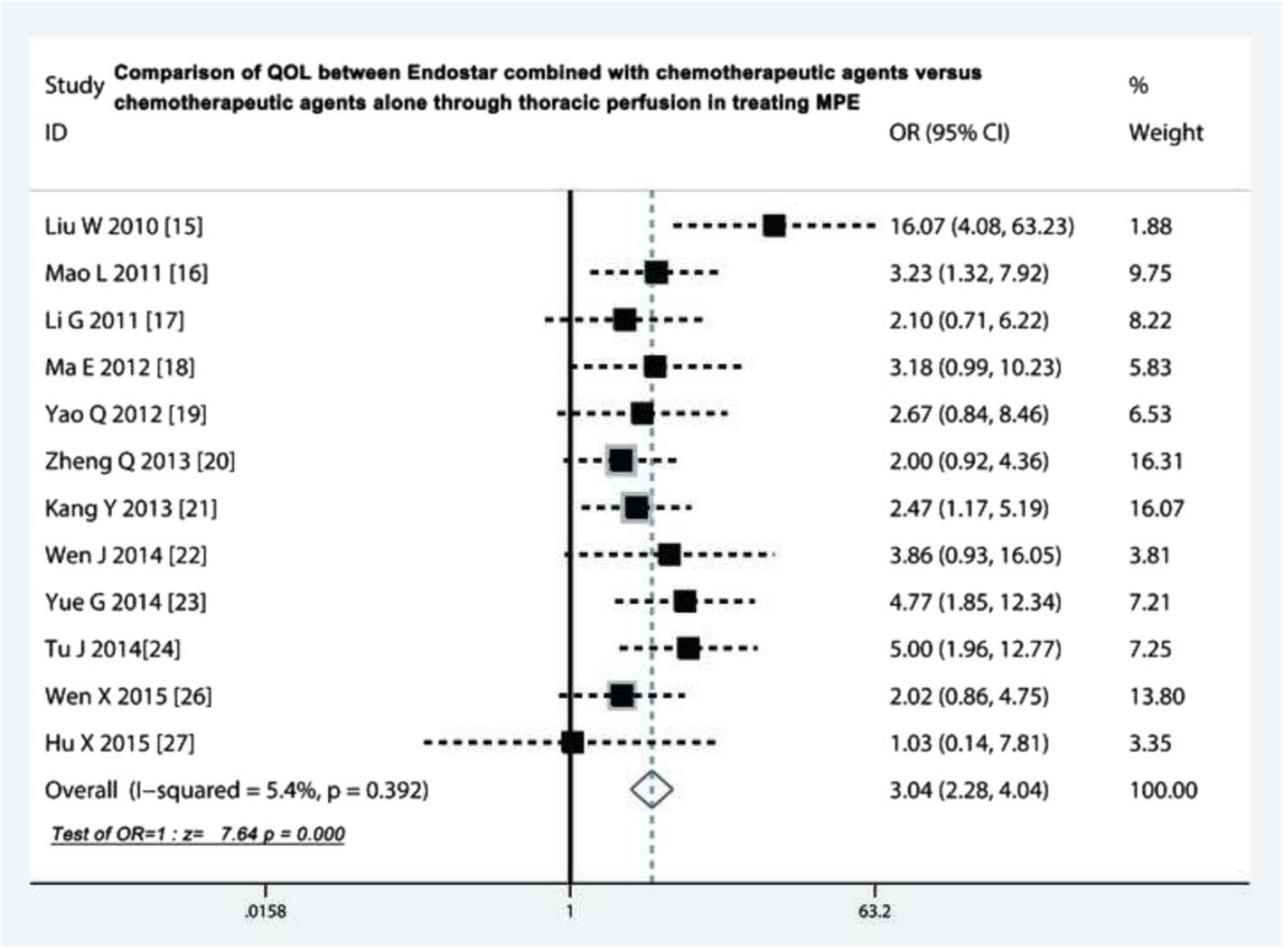
Comparison of QOL between Endostar combined with chemotherapeutic agents versus chemotherapeutic agents alone through thoracic perfusion for treating MPE. QOL, quality of life; OR, odds ratio; MPE, malignant pleural effusions

Twelve studies [15–24, 26, 27] investigated the changes of QOL after treatment. The Endostar combination arms had a higher symptom improvement rate than chemotherapeutic agents alone (odds ratio = 3.04, 95 % CI 2.28 to 4.04; test for overall effect: Z = 7.64, *p* < 0.001) (Fig. 4).

### Adverse reactions comparison of two projects

**Fig. 5:**
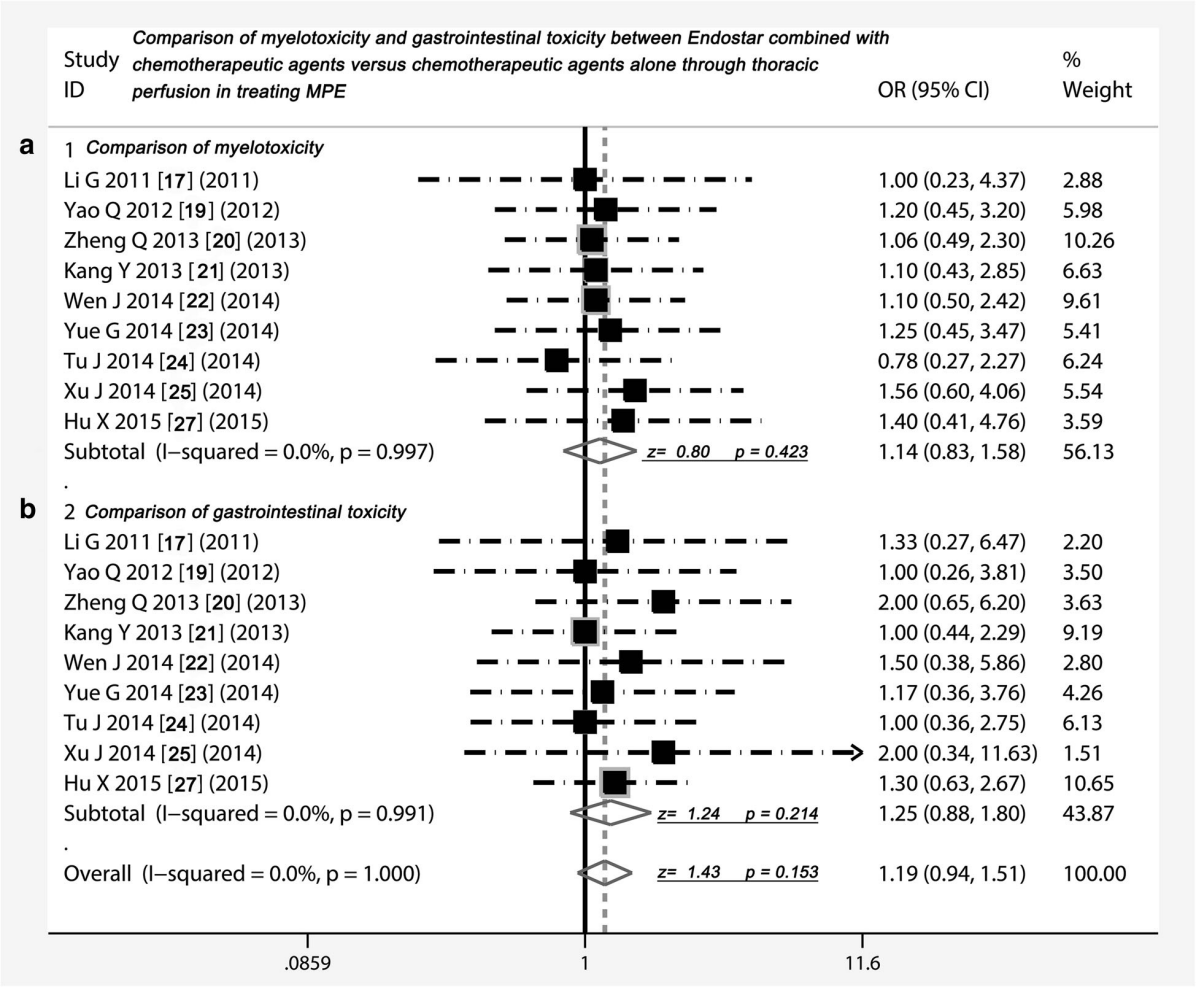
Comparison of myelotoxicity and gastrointestinal toxicity between Endostar combined with chemotherapeutic agents versus chemotherapeutic agents alone through thoracic perfusion for treating MPE. **a** Comparison of myelotoxicity between Endostar combined with chemotherapeutic agents versus chemotherapeutic agents alone; **b** Comparison of gastrointestinal toxicity between Endostar combined with chemotherapeutic agents versus chemotherapeutic agents alone; OR = odds ratio; MPE, malignant pleural effusions

**Fig. 6:**
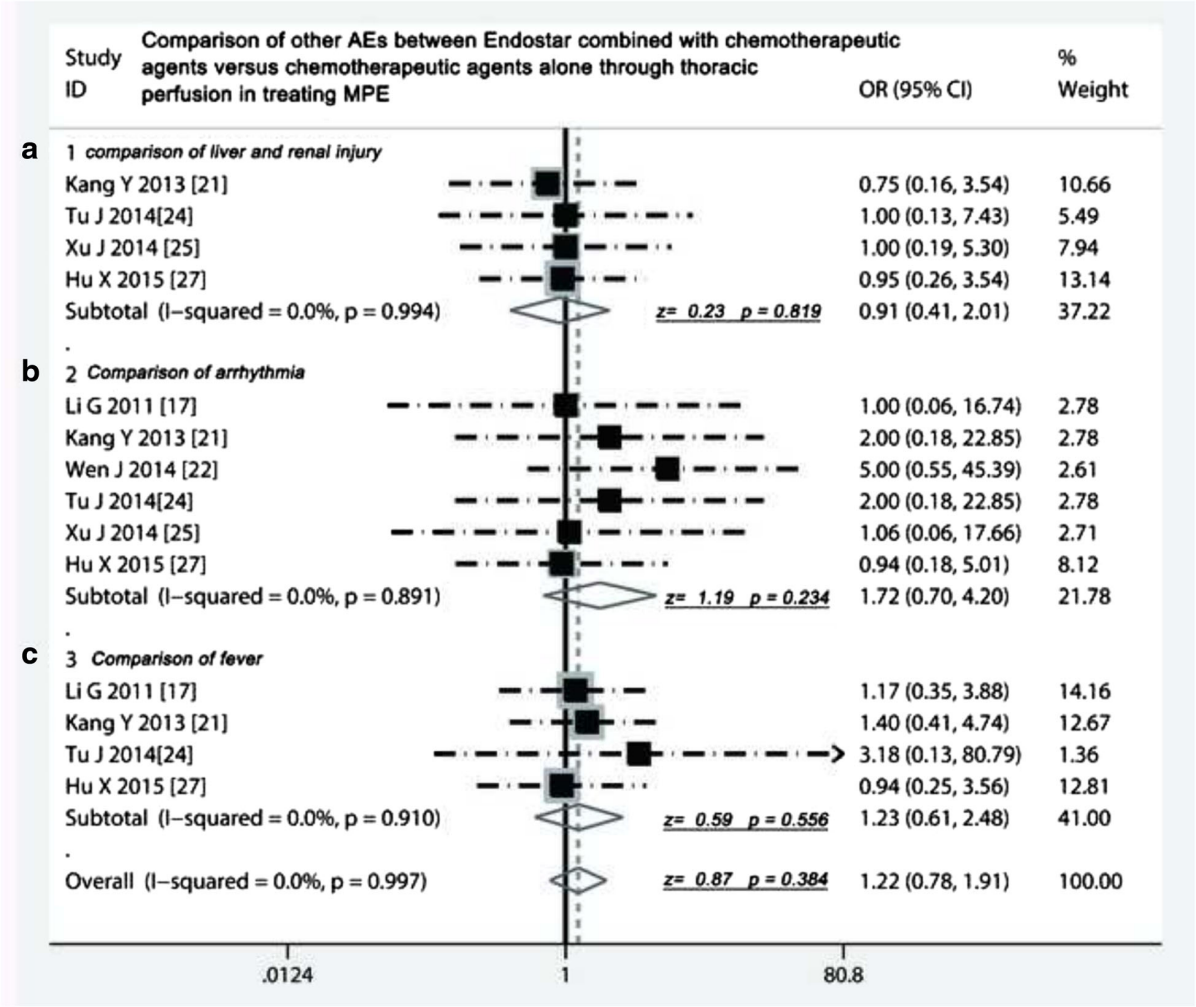
Comparison of liver and renal injury, arrhythmia and fever between Endostar combined with chemotherapeutic agents versus chemotherapeutic agents alone through thoracic perfusion for treating MPE. **a** Comparison of liver and renal injury between Endostar combined with chemotherapeutic agents versus chemotherapeutic agents alone; **b** Comparison of arrhythmia between Endostar combined with chemotherapeutic agents versus chemotherapeutic agents alone; **c** Comparison of fever between Endostar combined with chemotherapeutic agents versus chemotherapeutic agents alone; OR = odds ratio; MPE, malignant pleural effusions

As shown in Table 4, nine studies compared the adverse effects, which presented five common AEs including myelotoxicity, gastrointestinal toxicity, liver and renal function injury, arrhythmia and fever. The meta-analysis showed that incidence of myelotoxicity [17, 19–25, 27] were similar in Endostar combined with chemotherapeutic agents and chemotherapeutic agents alone (odds ratio = 1.14, 95 % CI 0. 83 to 1.58, *p* =0.423) (Fig. 5a). The incidence of gastrointestinal toxicity in two projects [17, 19–25, 27] did not have a significant difference (odds ratio = 1.25, 95 % CI 0. 88 to 1.80, *p* =0.214) (Fig. 5b). Five studies [17, 21, 24, 25, 27] compared liver and renal injury, six studies compared arrhythmia [17, 21, 22, 24, 25, 27], and four studies compared fever, all results suggested that the incidence rate of these AEs did not have differences between both of two projects (*p* > 0.05) (Fig. 6a, b and c).

**Table 4:** Comparison of adverse events between Endostar combined with chemotherapeutic agents versus chemotherapeutic agents alone

Study	Myelotoxicity (%)		Nausea/vomiting (%)		Liver and renal injury (%)		Arrhythmia (%)		Fever (%)	
	Group 1	Group 2	Group 1	Group 2	Group 1	Group 2	Group 1	Group 2	Group 1	Group 2
Li G 2011 [17]	4 (13.3)	4 (13.3)	4 (13.3)	3 (10)	–	–	1 (3.3)	0 (0)	7 (23.3)	6 (20)
Yao Q 2012 [19]	12 (40)	10 (30)	5 (16.7)	5 (16.7)	–	–	–	–	–	–
Zheng Q 2013 [20]	17 (28.3)	16 (26.6)	10 (16.7)	5 (8.3)	–	–	–	–	–	–
Kang Y 2013 [21]	11 (24.4)	10 (22.2)	15 (25)	15 (25)	4 (8.8)	3 (6.6)	2 (3.3)	0 (0)	7 (15.5)	5 (11.1)
Wen J 2014 [22]	22 (73.3)	20 (66.6)	6 (20)	4 (13.3)	–	–	5 (16.6)	1 (3.3)	–	–
Yue G 2014 [23]	10 (23.3)	8 (18.6)	7 (16.3)	6 (14)	–	–	–	–	–	–
Tu J 2014 [24]	7 (15.5)	9 (20)	9 (20)	9 (20)	2 (4.4)	2 (4.4)	2 (4.4)	1 (2.2)	–	–
Xu J 2014 [25]	14 (40)	9 (25.7)	4 (11.4)	2 (5.7)	2 (5.7)	1 (2.8)	1 (2.8)	1 (2.8)	1 (2.8)	0 (0)
Hu X 2015 [27]	7 (16.3)	5 (12.2)	26 (60.4)	20 (48.7)	5 (11.6)	5 (12.1)	3 (6.9)	3 (7.3)	5 (11.6)	5 (12.1)
	*P* > 0.05		*P* > 0.05		*P* > 0.05		*P* > 0.05		*P* > 0.05	

Values are given as number of patients (%). Group 1 = Endostar combined with chemotherapeutic agents; Group 2 = chemotherapeutic agents alone

### Assessment of publication bias and meta-regression analysis

**Fig. 7:**
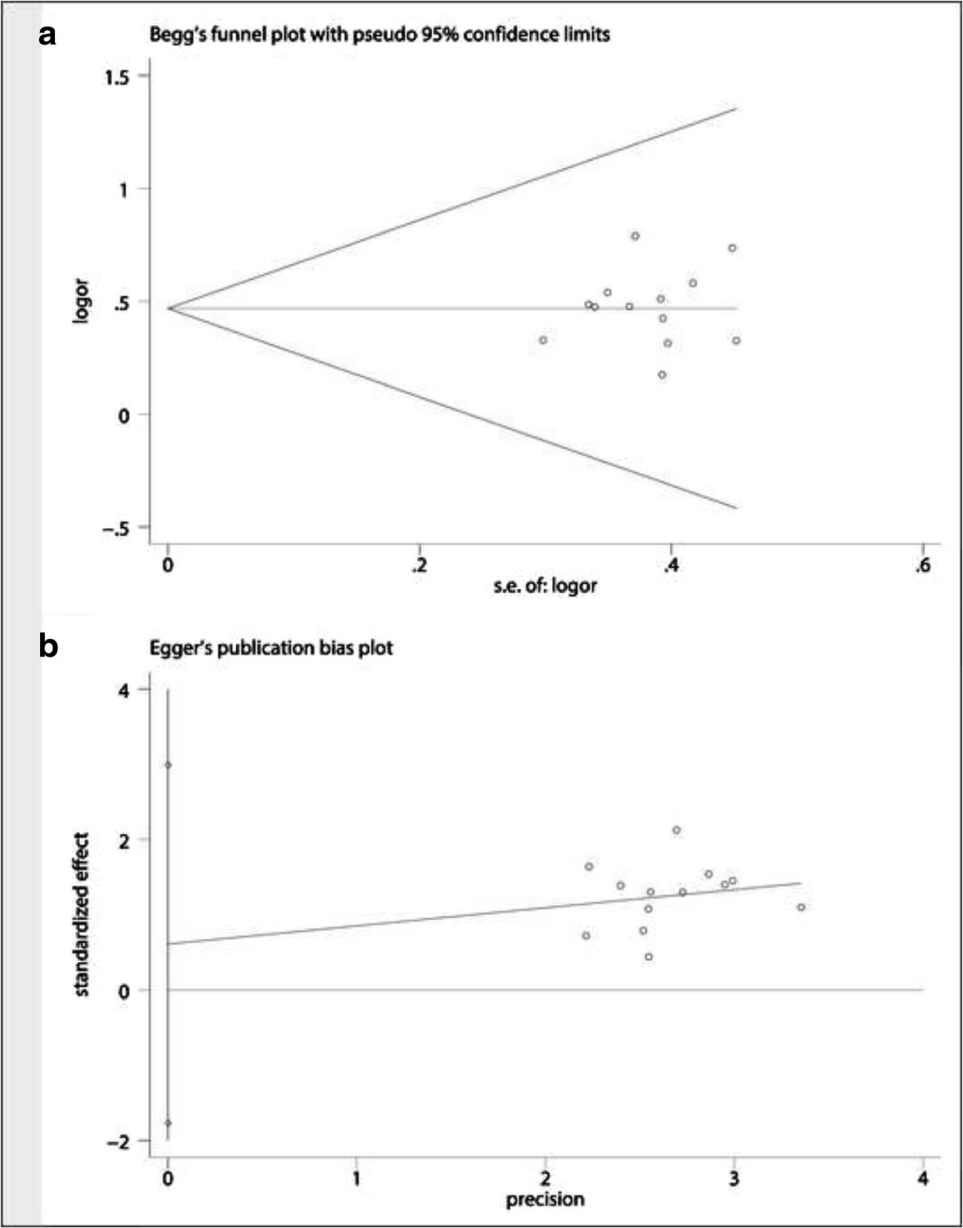
Assessment of publication bias. **a** Egger’s test did not imply a publication biases; **b** Begg’s test did not show the statistical significance

The shape of Begg’s funnel plot seems to be symmetrical (Std. Dev. of Score = 16.39, z = 0.37, Pr > z = 0.716), suggesting that publication bias did not have an impact on the results (Fig. 7a). The Egger’s test showed that t value was 0.60 with 12° of freedom (*P* = 0.562) (Fig. 7b). Get together, all evidence showed that no publication biases existed in these included studies. Test for heterogeneity of meta-regression showed that Q was 9.548 on 12° of freedom (*p* = 0.656), and moment-based estimate of between studies variance was zero (tau2 of size of sample = 0; tau2 of ITT = 0), which indicated that no obvious variation between groups was observed in this meta-analysis.

## Discussion

In clinical work, malignant pleural effusions (MPE) is a common problem that physicians, oncologists and thoracic surgeons often face. Although many malignant tumors directly lead to accumulation of pleural effusions, the mainly causes for MPE are lung cancer (37.5 %), breast cancer (16.8 %), and lymphoma (11.5 %). It is reported that 8 to 15 % of lung cancer patients presented symptom of MPE [30]. The local treatment was primarily current mode of administration for patients with MPE, including closed thoracic drainage, chemical pleurodesis and thoracic perfusion of antineoplastic agents such as doxorubicin, carboplatin, cisplatin, mitomycin C and 5-fluorouracil [31]. So far, a number of studies have reported on the advantages and security of Endostar combined with chemotherapeutic agents versus chemotherapeutic agents alone through thoracic perfusion for treating MPE. We summed up 13 RCTs and found that Endostar combined with chemotherapeutic agents through thoracic perfusion had better ORR and DCR benefits compared with chemotherapeutic agents alone (odds ratio = 3.58; 2.97 respectively) for treating MPE, translating into a 29 and 18 % absolute improvement respectively. These results corroborate that thoracic perfusion of Endostar take an active role in controlling MPE, which indicate that it is a new potential treatment alternative for treating MPE. Previous studies have demonstrated that Endostar inhibits endothelial cell migration, represses the neovascularization of new tumors, blocks the nutrient supply of tumor cells, and thus suppresses tumor proliferation or metastasis [32]. In addition, Endostar also could inhibit tumor lymphangiogenesis and reduce tumor cells into the bloodstream through the lymphatic [33]. More importantly, Endostar plays an efficient anti-cancer role in MPE through its suppressive effect on angiogenesis and lymphangiogenesis, suggesting that Endostar down-regulated the expression of VEGF-A and VEGF-C, thus inhibit the progression of MPE [34].

MPE is a common manifestation of disease progression to patient with advanced lung cancer and other cancers. In order to control symptoms and improve the quality of life, careful evaluation of pathology and patient treatment individualization is very crucial [2]. In addition to the cure of the primary disease, the improvement of QOL is important indicator of disease control, especially to malignant tumors. We all known that most of malignant tumor can not be cured, but can slow down the progression and ameliorate symptoms. Our meta-analysis showed that participation of Endostar remarkably improved the QOL of MPE (OR = 3.04, 95 % CI 2.28 to 4.04), which led to an absolute 29.1 % improvement of the QOL compared to chemotherapeutic agents alone. Previous study pointed out that Endostar suppress the VEGF-induced tyrosine phosphorylation of KDR/Flk-1 (VEGFR-2) as well as the overall VEGFR-2 expression and the activation of ERK, p38 MAPK, and AKT in human umbilical vein endothelial cells, which shows the relationship between Endostar and VEGF signal pathways and provide a molecular basis for the antiangiogenic effects of Endostar [12]. Also, Endostar can exert its anti-tumor effect via suppressing b-FGF-induced angiogenesis and b-FGF-activated MAPK signaling pathway, suggesting that Endostar might be a useful agent for treatment of malignant tumors [11].

We found that myelotoxicity and digestive reactions are most common adverse reactions, but most of which were grade 1 or 2 and were well tolerated. Through the further analysis, we noticed that the incidence of myelotoxicity, digestive reactions, liver and renal injury, arrhythmia and fever in treatment of Endostar combination was as high as that in chemotherapeutic agents alone, suggesting that the Endostar did not have an extra impact on the incidence of the AEs. The detection of heterogeneity is very important to meta-analysis, because it will affect the pooled statistical efficacy. We carefully assessed the included studies and found that those studies had a good clinical homogeneity. Moreover, the Egger’s test and the Begg’s test did not imply the possibility of publication bias.

However, there are some deficiencies in included trials. First, most of studies lack adequate analysis of subgroup data such as age, sex, smoking, histology, and treatment status and so on. Second, design quality of some is relatively low. Third, sample size of some is too small. The last, and mostly importantly, most of patients were from China (because Endostar was approved by the China State Food and Drug Administration and applied in treatment of lung cancer), which may lead to geographical and ethnic differences. In spite of this, these studies still propose a credible suggestion pointing toward that the Endostar is effective and safe for treating MPE, and it is a new choice for treating MPE. Nevertheless, Endostar, as a new molecular targeted drug, still needs to be investigated in the future. Especially, rigorously randomized control trials with large sampler size and multi-centered cooperation should be done before it could be recommended in clinic extensively.

## Conclusion

Thoracic perfusion of Endostar combined with chemotherapeutic agents has a better benefit of ORR and DCR for treating MPE and improves the QOL of MPE patients, compared with chemotherapeutic agents alone. Moreover, the participation of Endostar does not have an extra influence on the incidence of AEs. However, rigorously randomized control trials should be required before it is used widely.

## Abbreviations

AEs: Adverse reactions
AKT: Protein kinase B
CI: Confidence intervals
CNKI: China National Knowledge Infrastructure
DCR: Disease control rate
ERK: Extracellular regulating kinase
FGF: Fibroblast growth factor
KDR/Flk-1: VEGF receptor
MPE: Malignant pleural effusions
OR: Odds ratio
ORR: Overall response rate
p38MAPK: Mitogen activated protein kinases
QOL: Quality of life
RCTs: Randomised controlled trials
SFDA: China State Food and Drug Administration
VEGF: Vascular endothelial growth factor
